# Retracing the rabbit's path: Effects of altering the second flash position in the visual saltation illusion

**DOI:** 10.1177/20416695241254016

**Published:** 2024-05-21

**Authors:** Sheryl Anne Manaligod de Jesus, Hiroyuki Ito, Tama Kanematsu

**Affiliations:** Graduate School of Design, 12923 Kyushu University, Fukuoka, Japan; Faculty of Design, 12923 Kyushu University, Fukuoka, Japan; Faculty of Design, 12923 Kyushu University, Fukuoka, Japan; Center for Applied Perceptual Science, 12923 Kyushu University, Fukuoka, Japan

**Keywords:** visual saltation, cutaneous rabbit effect, visual illusion, perceptual grouping

## Abstract

Two dots shown in quick succession at one point and a third at a distance on the same linear path creates an illusion of intervening flashes on a visual field, a phenomenon known as the reduced visual rabbit illusion or visual saltation illusion. This study presents this illusion in a novel way by altering the position of the second flash, which has been typically presented only in the same position as the first flash. A series of experiments were conducted to observe whether saltation would occur if the second flash was presented in the same position as the third flash, out of sequential order relative to the first and last flash, or out of linear alignment at the midpoint between the first and the last flash. When all three flashes were presented in quick succession, participants misperceived the second flash to occur close to the midpoint between the first and last flash. Saltation was achieved in all three novel conditions, hinting a particular neurological process may be responsible for shared outcomes.

The human eye's detection of a stimulus position is not reliable under certain conditions. The visual rabbit illusion, also called the visual saltation illusion (VSI) demonstrates this disparity (Geldard, 1976). Under the right settings, two flashes shown in the same position followed by a third flash at distance, are perceived to occur in succinct order, with the second flash mislocalized to occur at the midpoint between the first and the third flash. This phenomenon is the visual modality of the cutaneous rabbit effect (CRE), one of the first experiments that showed that the sense of touch can be tricked into perceiving “hops” across the skin equidistant to each other between the first and final stimulus points even though such loci were never stimulated (Geldard & Sherrick, 1972).

As the CRE highlighted that physical reality does not align with perception, researchers applied the same paradigm to other modalities. When reproduced through the sense of hearing it confirmed that cutaneous and auditory perception share the same manner of misinterpreting information (Bremer et al., 1977). And, although the parameters were different, short interstimulus intervals (ISIs) and the sequential timing in pulses were found to be important to misguide the senses (Bremer et al. 1977). Since then, the CRE has been studied in the somatosensory modality (Blankenburg et al., 2006) and even projected “out of the body (Lee et al., 2012; Miyazaki et al., 2010).” Thermoceptive and nociceptive pathways were also subject to the illusion even at slower ISIs compared to Geldard's (1976) original experiment (Trojan et al., 2006). Although a hopping sensation can still be perceived cutaneously if visual attention is not directed on stimulation points on the skin (Flach & Haggard, 2006), cross-modal studies also reveal the impact vision has on the CRE. When an observer views flashes that are congruent with the timing with the typical illusionary CRE taps, the CRE is reported to feel much stronger, while changing timing of flashes reduces the effect greatly (Asai & Kanayama, 2012). As for the vision modality, saltation typically occurs only peripherally (Geldard, 1976; Goldreich & Tong, 2013) as described below. This limitation may be the reason the saltation effect in vision has not had as much variation in research versus tactile and auditory studies.

A reduced version of the CRE commonly tested today utilizes only three stimuli at two locations, with the first and second stimuli occurring in the same spot. Geldard (1976) carried out a reduced VSI using flashing lights. His study emphasized the importance of retinal position relative to stimuli and the fixation point, describing the ideal visual eccentricity for stimuli at a position 25 to 30 deg from the fovea, while a visual eccentricity of 50 deg could still induce the illusion but with more difficulty. Geldard (1976) also reinforced saltation illusions’ feature of favoring short ISIs, where ISIs below 100 ms can cause a flash to “leap” at longer distances from the first flash position (L1) to the midpoint (L2). ISIs approaching 300 ms would induce only leaps of 10% or none at all (Geldard, 1976). The constraints on retinal eccentricity and timing are reminiscent of the fact that this phenomenon is based on lower-level processing. However, Lockhead et al. (1980) built on the VSI following the parameters of Geldard and Sherrick (1972) and showed that subjects perceived flashes to occur within their blind spot, an area where visual stimuli should not be detected. This gave further insight into how the brain processes the VSI stimuli. Since then, different visual aspects have been examined under the VSI, such as Khuu et al. (2010) using 3D images and Ito et al. (2023) using Kanizsa-type subjective contours. Such studies revealed that the illusion still holds strength even if the physical aspects of stimuli increased in complexity.

Motion-based phenomena have been cited as possible explanations for the VSI. The Fröhlich effect (Fröhlich, 1924) causes the perceived shift of the starting position of a moving object in the direction of its motion. MacKay (1958) found that putting slight pressure on the eyeball can cause a viewer to observe disparity in apparent motion between self-luminous and stroboscopically lit surroundings. Nijhawan (1994) re-discovered the Mackay's phenomenon, which has become well known as the flash-lag effect. That is, a moving object is perceived to go ahead of a flashed object even though both stimuli were physically aligned when the flash appeared. In the motion drag illusion (Whitney & Cavanagh, 2000), the position of a flash is misperceived, shifting to the direction of nearby motion. The flash drag effect is caused not only by real motion but also by bistable apparent motion (Shim & Cavanagh, 2006). In the case of VSI, apparent motion between the first (or second) flash and the third flash could be hypothesized. The motion-based position-shift phenomenon generally shifts the perceived position of a flash to the forward direction of motion. This matches the perceived shift of the second flash position in VSI.

Another explanation for the VSI is not based on motion signals. Shore et al. (1998) noted that the auditory saltation illusion could be explained by perceptual grouping. The perceptual system seems to prefer a simple interpretation for ambiguous or complicated stimuli, according to the principles of Gestalt psychology. Since swift presentation of flashes makes it difficult for observers to exactly perceive the positions of the flashes, they may select the simplest interpretation of the spatial relationship of the stimuli. In that process, a later event could affect the interpretation of the earlier event. This hypothesis is called postdiction (Eagleman & Sejnowski, 2000; Shimojo, 2014). When the three flashes are presented within a certain temporal window, after the third flash physically appears, the interpretation of the group of events (three flashes) could be constructed.

This study was conducted to further examine potential causes of the VSI by using novel second flash positions. This paper tests the low-level motion-signal based explanation of the VSI and demonstrates a new type of VSI that has not been reported to date. Varied positions of the second flash were used as main factors in three experiments. The differences and similarities in responses throughout all three experiments will reveal whether there is shared mechanism in position misperception.

In Experiment 1, the second flash was presented at the same position as the third flash. Asai and Kanayama (2012) had tested this backwards presentation (L1-L3-L3) with the CRE and showed that, although less frequently than the typical forward CRE pattern (L1-L1-L3), subjects can perceive taps at three separate locations (L1-L2-L3). Pairing the backwards presentation with a flash that is congruent to the midpoint of the first and third tap can strengthen this perception as well. However, Asai and Kanayama (2012) did not require subjects to locate the position of the second tap, nor was any response collected regarding the flash stimuli. Would same results occur solely using visual stimuli? We hypothesize the perceptual position shift of the second flash would not arise if VSI is caused by motion signals because there are only possible motion signals arising between the first and the second (or the third) flashes and there is no room for a forward position shift of the second flash. To perceive the hopping, that is, to perceive the second flash as to be in midway of the first and the third flash positions, the position of the second flash should shift backward. Thus, the stimulus condition could test the motion-based explanation of VSI.

In Experiment 2, the motion-based hypothesis was further investigated by breaking the spatio-temporal relationship through reversed conditions, where the second flash appeared outside the area between the first and the third flashes. The direction of a hypothesized motion signal between the first and second flashes was opposite to that between the second and third flashes. Furthermore, the positional relationship was not sequential here. The most similar phenomena akin to this second flash presentation would be the flash grab effect, a motion-induced effect that does not rely on motion in one continuous direction. A texture presented repeatedly moving in one direction and then reversed can induce a positional shift of a flash shown at the moment of the motion direction reversal (Cavanagh & Anstis, 2013). Although the VSI is not presented on a moving background, the presentation of the second flash out of bounds can be likened to the back-and-forth motion of the flash-grab. Since “motion” of the VSI would reverse at the second flash position, it may account for perceiving the second flash close the flash (either the first or the third) it was presented to, those but may not be sufficient to explain perception at the midpoint.

In Experiment 3, the second flash was presented midway between the first and the third flashes but with a physical shift in a right-angle direction. In the grouping hypothesis, the second flash position should not be limited to being aligned with the first and the third flashes to cause VSI. If the hopping-in-line percept arises even when the position of the second flash shifts from the midway point between the first and the third flashes in any direction, the VSI may not be based on low-level motion signals but caused in a higher-level interpretation process.

## Experiment 1: Second Flash in the Backward Shift Condition

As aforementioned, the VSI is typically presented with the first two stimuli in the same position. The objective of this experiment is to observe if results can be replicated if the experiment were to be presented backwards, or where the last two stimuli are presented in the same position. Preliminary tests were conducted to observe whether all three stimuli can be perceived even when the last two stimuli were presented in the same position. All three stimuli were perceived in the correct sequence, with the first and third flashes being perceived approximately at the actual flashed positions, and the second flash perceived to occur at a point between. Since perception of the first and third flash locations were established to be consistently identified between participants, the following experiment asked participants to identify the second flash only.

### Methods

#### Participants

Thirty-nine participants (18 men, 17 women, and four who did not disclose their gender, ranging in age from 18 to 34 years) with normal or corrected normal vision participated in this experiment, eight of whom were familiar or aware of the VSI. All participants were informed of the possible risks, gave written consent, and were compensated monetarily for their time.

#### Apparatus

The PsychoPy (Peirce et al., 2019) program was used to create the experiment, which was displayed on a 24.5-inch organic light-emitting diode display (SONY PVM- 2541) in a dark room (Ito et al., 2013). The screen resolution was horizontally 1920 × vertically 1080 pixels. Participants’ eyes were distanced approximately 40 cm from the screen with their heads resting on a chinrest. The display refreshed at 60 Hz.

#### Stimuli

**Figure 1.**
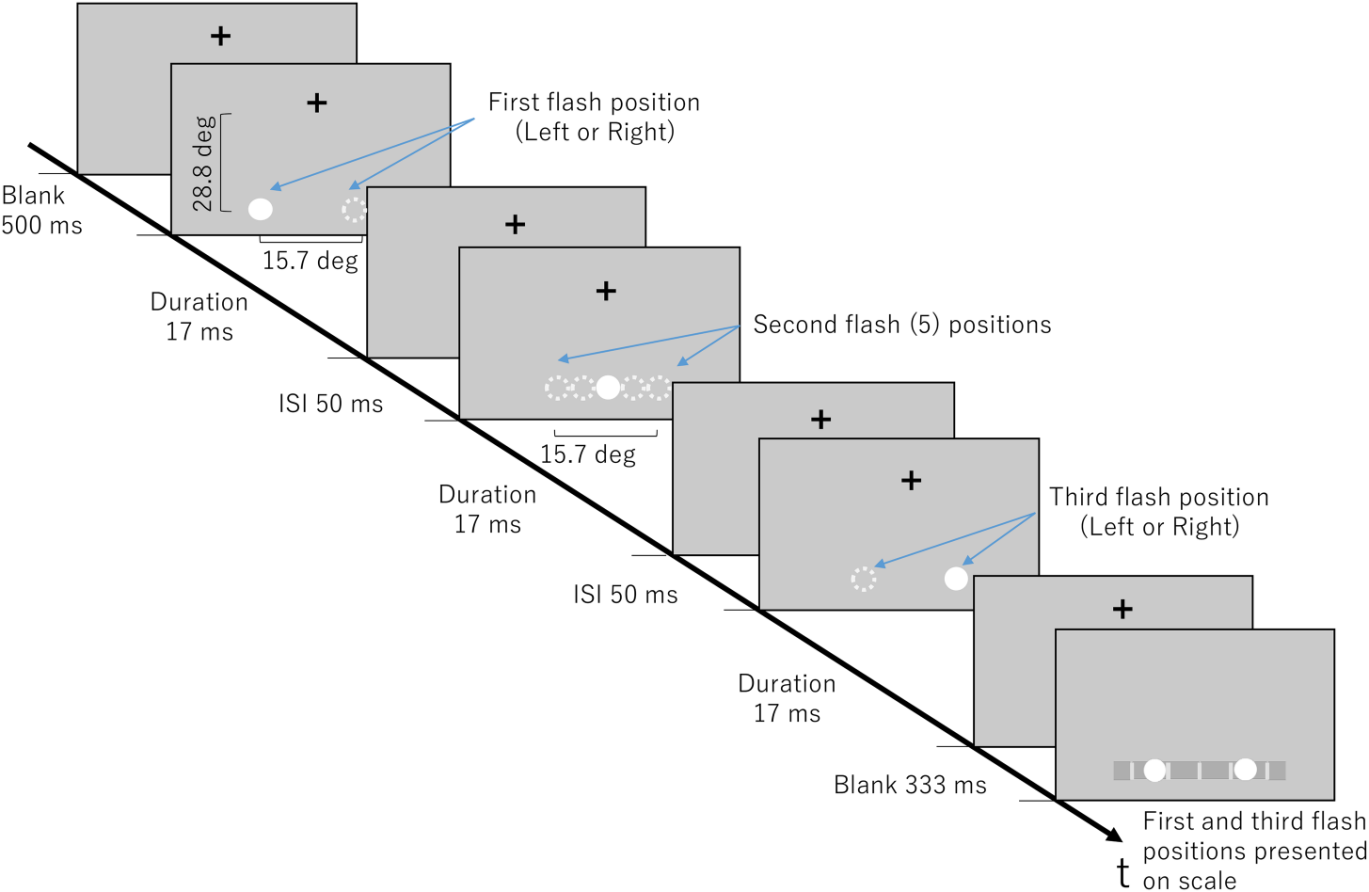
Experiment 1 stimulus parameters for the illusion condition. Experimental display that gives an example of stimuli moving in the left to right direction, indicated by solid white circles. Dashed white circles indicate other possible positions the flash could occur during the indicated time.

Flashes were white circles, measuring 100 pixels (4.1 deg) in diameter with a luminance of 99.4 cd/m^2^ and presented on a grey background that had a luminance of 19.8 cd/m^2^. The first and the third flash positions were presented 15.7 deg apart on the same horizontal line. Using the length of the first and third flash as a scale, the second flash was presented at the same position as the first flash (0%), at the midpoint (50%) between the first and third flash positions, at the center point between the midpoint and the first flash (25%), at the center point between the midpoint and the third flash (75%), and at the third flash position (100%) (Figure 1). Flashes originated from the left or right.

Stimuli had an ISI of 50 ms and a SOA (Stimulus Onset Asynchrony) of 67 ms in the illusion condition and an ISI of 950 ms and SOA of 1000 ms in the control condition. To investigate the effect of second flash position in the saltation illusion, a duration and ISI (thus, SOA) that could produce a clear saltation illusion was needed. Through a preliminary experiment, the short ISI and duration as noted above for the illusion condition was selected. Ito et al. (2023) also showed short SOAs are favored by the VSI. To demonstrate that the valid position could be perceived when the saltation illusion did not occur, the long ISI and duration as noted above were chosen for the control condition.

#### Procedure

Participants performed practice trials until they were comfortable with the task before commencing the actual experiment. They were instructed to fixate their eyes on a cross on the screen that was in horizontal alignment with their eyes during the trials. This fixation point was located 28.8 deg above the horizontal midpoint where the flashes would occur. A trial consisted of three flash stimuli. Participants were informed of the first and third flash locations on the monitor and that they will interchange randomly between trials; they were not informed of the exact second flash positions. After one trial, a scale would appear, and participants were instructed to click at a point on the scale where they perceived the second flash relative to the first and third flash.

The scale appeared in the same horizontal location as where the flashes occurred, as a gray bar with five light gray markings at equally spaced positions that did not correspond to any of the second flash positions, except for a mark at the center that represented the midpoint of the first and third flash positions as shown in Figure 1. White circles (same as the flash stimuli) on the actual position of the first and third flashes also appeared on the scale (Figure 1). Participants were advised that the white circles indicate the actual positions of the first and the third flash and can assist in identifying where they perceived the second flash. They were also advised the white markings could help to make their selection more precise, but that the markings did not represent locations of flashes. Participants were told to click anywhere on the scale where they perceived the second flash. After a selection was made, the next trial began.

Each participant underwent the 10 conditions (5 s-flash positions × 2 directions) six times in random order under the illusionary condition and the control condition, resulting in two blocks of 60 trials for a total of 120 trials. The administration order of the illusion and control block was randomly assigned for each participant to observe for any order effects.

#### Data Analysis

R software (2021) was used to analyze perceived positions of the second flash relative to the position of the first and third flash. Three participants’ responses were not included in the final data analysis after noticing their results had abnormalities in the control conditions. In control conditions, two participants reported the second flash to occur at the opposite location. For instance, if the second flash was presented in the same location as the first flash, the two participants would report the second flash to occur in the same area as the third flash. If the second flash was presented 25% from the third flash, they would report it to occur 25% from the first flash. One of these participants later reported that they underwent brain surgery but was not aware that this affected their vision, which could account for their particular response patterns. The third participant reported mostly seeing one flash, rarely two or three, in illusionary conditions and would typically select the position of where they perceived the first flash. Under control conditions, this participant reported seeing mostly two to three flashes, and would typically select the first or their flash positions as where they perceived the second flash. Due to the inability to perceived approximate locations of the second flash in control conditions their data was not included and, the results are that of 36 participants only.

The five physical second-flash positions and corresponding perceived second-flash positions were aggregated with horizontal reversals according to the presentation direction (left-to-right or right-to-left). Clicking the center mark of the scale would be equivalent to a proportional value of 50%, while clicking at the exact point of the first flash position and third flash position would be equal to 0% and 100% respectively. These proportional values were used for data analysis. Preliminary tests showed that gender and age did not make a substantial difference in the overall results.

### Results and Discussion

**Figure 2.**
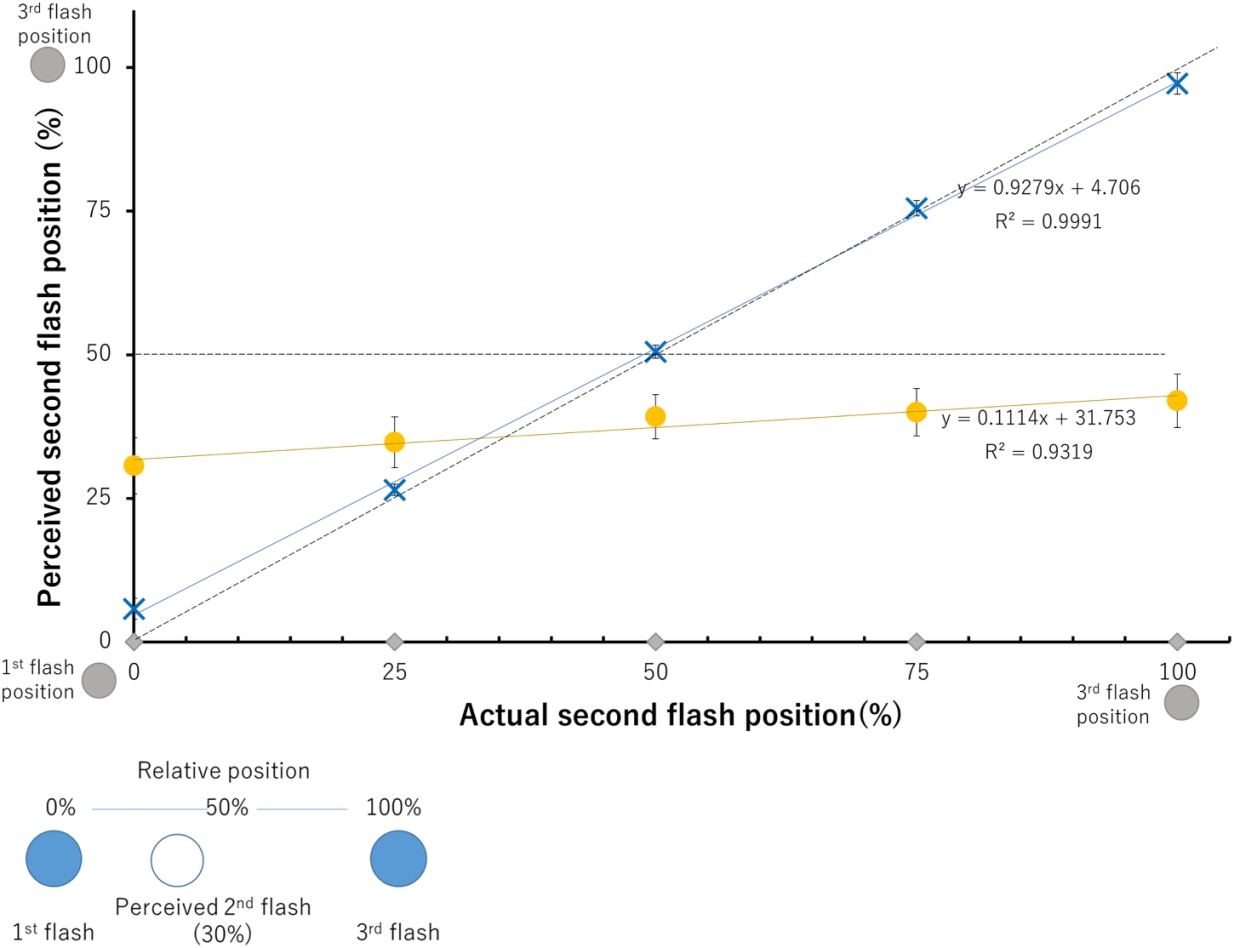
Perceived proportion of second flash positions relative to the first and third flash. Grey diamonds on the horizontal axis indicate actual second flash positions, with 0 representing the second flash in the first flash position and 100 representing the second flash in the third flash position. Yellow circles indicate participant responses in illusion conditions corresponding to the five second flash positions. Blue x's indicate participant responses in control conditions corresponding to the same five positions. The dashed diagonal line indicates values where the second flash would be perceived at its physical position, while the horizontal dashed line indicates saltation perceived at the midpoint. Error bars indicate standard errors of means (SEs). The inset provides an example of what the numerical values of the *y*-axis indicate in the main graph.

Figure 2 displays the proportions of perceived second flash positions relative to the first flash location for both illusionary and control conditions across 36 participants. As data analysis revealed the effect of direction was not significant; Figure 2 shows the combined responses from the left and right directions. Consistent with previous studies, for illusionary conditions where the second flash was presented in the same position as the first flash position (0% for the horizontal axis), the perceived second flash position was around 30%, that is, the saltation illusion occurred (Figure 2). Presentation of the second flash in the same position as the third flash led participants to mislocalize the flash 1.0–0.5 deg (11.8–21.2%) from the center point of the first and third flash, achieving saltation. The perceived second-flash positions did not change greatly despite the actual position changes between the first and third flash positions. For control conditions, the second flash was perceived to occur close or approximately at the actual position it was flashed, indicating that regardless of the second flash position, duration and timing of the stimuli are what influence the perception of hopping across a screen. The results indicate a VSI is perceived with a postdictive position change of the second flash under varied second flash conditions.

A three-way repeated measures analysis of variance (ANOVA) was conducted to test the main effects of direction, the position of the second flash, and the presentation timing (illusion or control), as well as their interaction effects on perception of the second flash. As the violation of sphericity was indicated by the Mauchly's test, Greenhouse-Geisser's epsilon was used to adjust the degree of freedom. The effect of direction of the flashes was not significant (*F*(1,35) = 0.5961, *p *= .4453, *ηp^2 ^*= 0.0167). The presentation timing factors (*F*(1, 35) = 16.795, *p *= .0002, *ηp^2^*= 0.3239) and the effect of positions of the second flash (*F*(3.17, 110.79) = 343.5689, *p *< .0001, *ηp^2^*= 0.9705) were highly significant. The interaction effect between direction and the presentation timing as factors was significant (*F*(1,35) = 6.1823, *p *= .0178, *ηp^2 ^*= 0.1501), suggesting perception of the second flash is somewhat influenced by the direction flashes were presented depending on the presentation speed. The interaction between the second flash position and presentation timing (*F*(3.04, 106.5) = 192.5930, *p *< .0001, *ηp^2^*= 0.8462) was highly significant. This interaction is clearly seen in Figure 2, which shows the difference in regression coefficients between the control (0.9279) and illusion (0.1114) conditions.

Shaffer's Modified Sequentially Rejective Bonferroni Procedure (MSRBP) was utilized for pairwise comparisons between perceived flash positions in illusion and control condition responses. Under illusion conditions, significant differences in responses were found between the second flash at 0% and at 100% (*p* < .0001, adjusted *p* = .0002), 75% (*p* = .0002, adjusted *p* = .0013), and 50% (*p* = .0044, adjusted *p* = .0264); along with the second flash positioned at 25% and 100% (*p* = .0047, adjusted *p* = .0281). These reflect the shallow slope in the graph for the illusion condition (regression coefficient of 0.11). However, even under the 100% (at the third flash position) condition, the averaged perceived second flash position was under 50%, that is, closer to the first flash position than the third flash position. Under control conditions, significant differences were found between each flash position with higher significance levels; indicating that the difference in responses reflects the five different flash positions, together with the regression coefficient of 0.93 and *R*^2^ of almost 1.0.

The common neural mechanisms cited to explain the VSI, such as the Fröhlich effect (Fröhlich, 1924) and flash drag effect (Whitney & Cavanagh, 2000) are not sufficient to explain saltation when the second flash is presented in the same position as the third flash. If so, then the second flash would have been perceived to occur in the same position as or after the third flash, and the third flash would have also been perceived to have shifted toward the direction of movement. The similar perception of the second flash throughout the different actual flash positions (Figure 2) can reflect hypotheses that the brain makes a probabilistic assumption in order to make the most sense of stimuli that is not easily detectable (Goldreich & Tong, 2013; Shore et al., 1998). Perhaps the presentation of the flashes occurred at such high speeds that made it difficult for the brain to correctly process the second flash location. As a result, it is possible that throughout the illusion condition (flashes presented at a faster speed), the brain recounts the second flash to occur in the same spot in each direction respectively because it cannot detect the second flash accurately. In the control condition, when the flash is presented at slower speeds, the second flash can be identified and therefore correctly processed and reported, so there is no need to make a probabilistic assumption. This also indicates duration and ISI are important variables to make the illusion successful since the second flash in the control block was not misperceived.

Perceived second flash positions among participants who were administered the control block first were occasionally more accurate than those who were administered the illusion block first in some second flash positions. However, when analyzing their responses, no significant differences were found. This might be attributed to a possible practice effect resulting from exposure to the actual flash positions in the control conditions, which participants then applied to their responses in the illusion condition. To prevent this, the illusionary block was presented first for Experiments 2 and 3. Successful saltation of the second flash when presented at the same position of the third flash showed that similar parameters can also be applied to Experiment 2.

## Experiment 2: Second Flash in the Reversed Shift Condition

**Figure 3.**
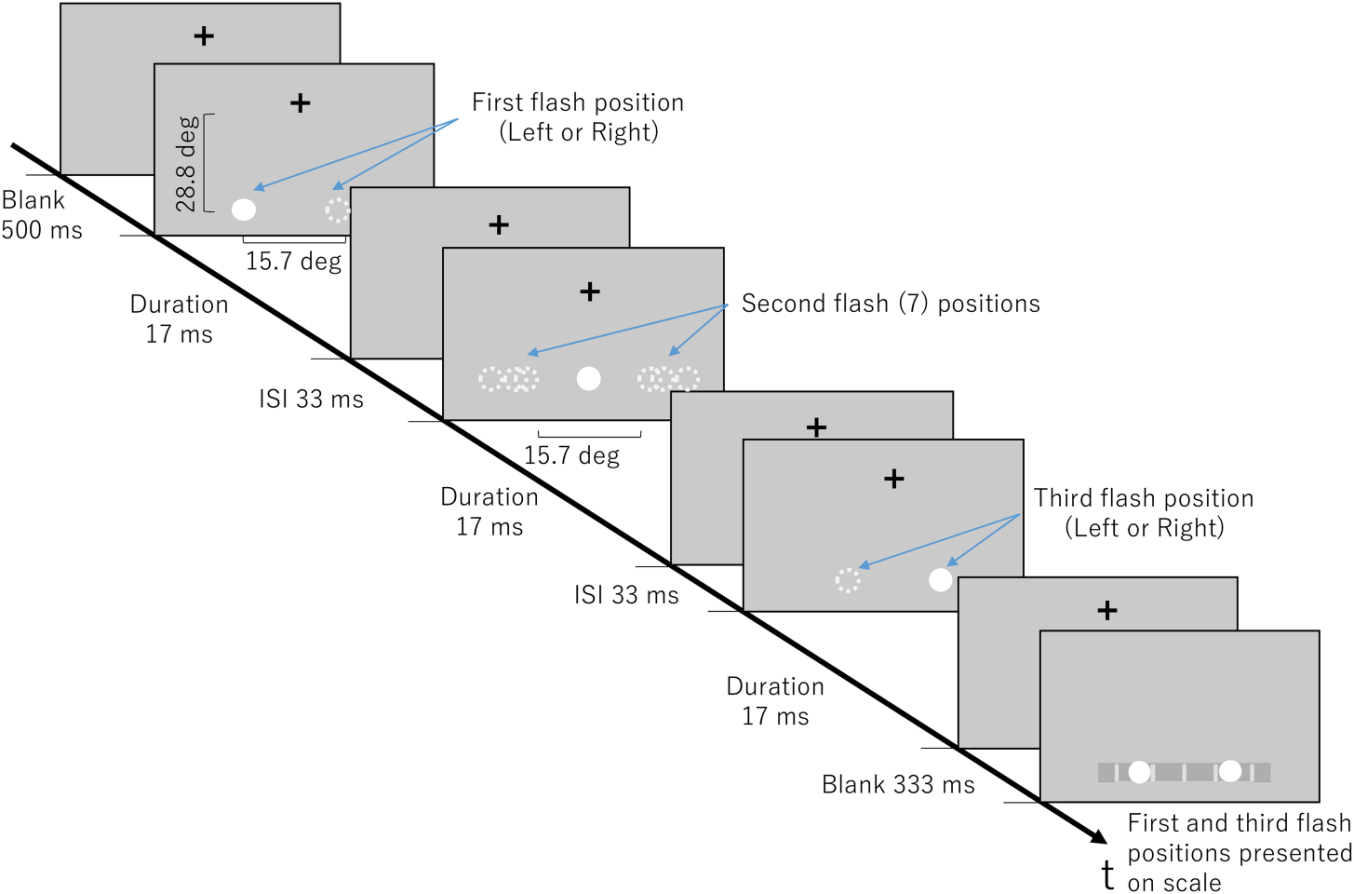
Experiment 2 stimulus parameters for the illusion condition. Experimental display that gives an example of stimuli moving in the left to right direction, indicated by solid white circles. Dashed white circles indicate other possible positions the flash could occur during the indicated time.

Experiment 2 aims to observe where the second flash is perceived when it is presented out of bounds of the first and the last flash, reversing the usual forward or backward sequence of stimulus presentations. Positions (Figure 3) that occurred out of bounds from the first or last flash were chosen. We hypothesize that second flash positions that occur closer to the first or last flash will be perceived closer to the midpoint of the first and last flash. The farther out of bounds the second flash is presented from either the first or third flash, the more likely a break in the flow of flashes will be perceived, making it less likely for the brain to formulate a consistent pattern.

Preliminary tests were done using the same stimulus parameters (ISI and SOA) as Experiment 1; however, the illusion was not always perceived, and could be considered weak. Preliminary participants were more likely to perceive the out-out-of-bounds positions close to their approximate positions. To demonstrate the illusion, the ISI and duration were decreased for these experiments. However, this sacrificed the ubiquitous characteristic of the experiment; under certain conditions, some could not perceive all three flashes. Only individuals who could perceive all three flashes during practice trials and distinguish the difference between two, three, and four flash stimuli participated in Experiment 2.

### Methods

#### Participants

A screening test was performed on participants before the actual experiment to ensure their vision was normal and that they could perceive three flashes throughout the trials. Screening tests included presenting trials with two to four flash stimuli, and a potential participant must be able to distinguish between the different number of presented flashes. Twenty-two university students (8 male, 13 female, and one person who did not disclose their gender, ranging in age from 20 to 31 years) with normal or corrected normal vision participated in this experiment. Ten were familiar with the VSI, seven of which participated in Experiment 1. Participants were informed of the possible risks, signed a consent form, and were compensated for their time. A co-author was a participant and did not receive compensation.

#### Apparatus, Stimuli, and Procedure

The apparatus and procedure were the same as Experiment 1. Stimulus appearance was also the same—only the positions of the second flash, stimulus duration, and ISI changed. The second flash was presented at the center of the first and the third flash or at positions that occurred outside the first or the third flash for a total of seven different flash positions (Figure 3). Participants were not advised of actual second flash positions but were told they could click at any point on the scale of where they perceived the second flash relative to the first and third flash. In terms of perceived proportion of the second flash relative to the first and third flash, with the value of 0% being the first flash and the value of 100% being the third flash, the second flash was positioned at values of −16%, −8%, −4%, 50%, 104%, 108%, and 116% relative to the first and third flash. Stimuli flashed from the left or the right, creating a total of 14 conditions. Participants underwent the 14 conditions six times in random order under an illusion block and a control block. The illusionary condition stimuli had an ISI of 33 ms and a SOA of 50 ms; control condition stimuli had an ISI of 33 ms and an SOA of 983 ms. Participants were administered the illusionary block first then were asked questions to verify the number of flashes they perceived before proceeding to the control block. Participants underwent 84 trials per block for a total of 168 trials.

### Results and Discussion

One participant's results were discarded based on their responses in the control conditions. Like the abnormal responses in Experiment 1, this participant would report perceiving the second flash “opposite” to its actual flashed position. For example, if the flashes were being presented in a right to left direction, and the second flash was presented two deg right of the first flash, they perceived the second flash two deg left of the third flash. The final data analysis includes the results of 21 participants only.

**Figure 4.**
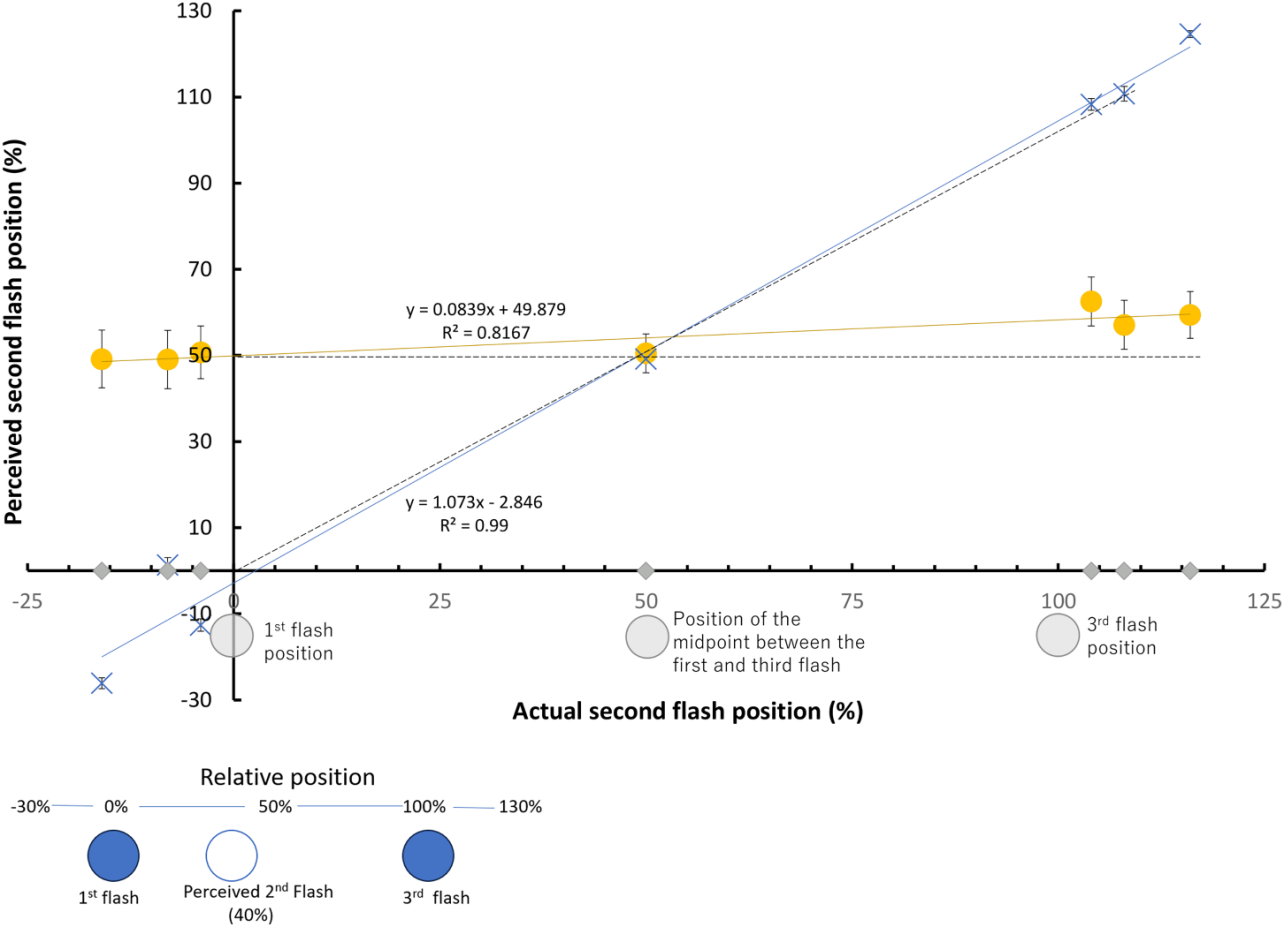
Perceived proportion of second flash positions relative to the first and third flash. Grey diamonds indicate actual flash positions, yellow circles (participant responses in illusion condition) and blue x's (participant responses in the control condition) correspond to the same positions. The dashed diagonal line indicates values where an illusion is not perceived, while the horizontal dashed line indicates saltation perceived at the midpoint. Error bars indicate standard errors of means (SEs). The inset on the bottom provides an example of the what the numerical values of the *y*-axis indicate in the main graph.

Figure 4 shows the results of the perceived proportion of the second flash position relative to the first and the last flash; responses in both direction presentations (right-to-left and left-to-right) are combined. On average, participants’ responses under illusion conditions were similar throughout the 14 conditions; reflected by the yellow circles that are almost parallel to the horizontal dashed line shown in Figure 4. Under the illusion conditions, the perceived second flash positions were almost constant around 50% against the changes in actual flash positions, demonstrating that the VSI occurred even when the second flash was presented out of spatial order. Blue Xs indicate the perceived proportion of second flash positions under control conditions. Control responses almost linearly reflected the actual flash positions even outside the horizontal range between the first and third flash positions. This means that participants could validly perceive the spatial positions of the flashes when the ISI and/or duration was long.

A three-way repeated measures ANOVA was conducted to observe the same three effects as Experiment 1. As the violation of sphericity was indicated by the Mauchly's test, Greenhouse–Geisser's epsilon was used to adjust the degree of freedom. The main effect of direction (*F*(1, 20) = 0.3498, *p *= .5609, *ηp^2^*= 0.0172) was not significant; participant responses did not vary between flashes presented in the left or right direction. The effect of stimulus presentation timing (*F*(1, 20) = 0.9793, *p *= .3342, *ηp^2^*= 0.0467) was not significant. The main effect of the second flash position was highly significant (*F*(3.71,74.18) = 991.0215, *p *< .0001, *ηp^2^*= 0.9802). The interaction effect between the flash position and presentation timing was found to be highly significant (*F*(3.54, 70.77)= 593.0878, *p *< .0001, *ηp^2^*= 0.9674). These statistics confirm that the perceived second flash positions were not much varied by the actual flash positions under the illusion conditions (regression coefficient was 0.0839) while the perceived flash positions strongly reflect the actual stimulus positions under the control condition (regression coefficient was 1.073 and *R*^2^ was 0.99).

Using the same post-hoc analysis as Experiment 1, in control conditions, MSRBP revealed significant differences in responses between all flash positions. The ability to distinguish the correct second flash positions when presented at slower speeds can be responsible for these differences, similar to Experiment 1. In illusion conditions, significant differences were found between the second flash position at −4% and at the midpoint (50%) (*p* = .0014, adjusted *p* = .0209), at 104% (*p* = .0012, adjusted *p* = .017), 108% (*p* = .0002, adjusted *p* = .0031), and 116% (*p* = .0001, adjusted *p* = .0031). The second flash position at −16% had significant differences between the flash positions close to the third flash position; 104% (*p* = .0022, adjusted *p* = .0327); 108% (*p* = .0007, adjusted *p* = .0108). Lastly a significant difference was present between the second flash positions at −8% and at 108% (*p* = .0013, adjusted *p* = .0188). It can be inferred that the second flash positions close to the first flash (−16%, −8%, −4%) may be perceived in a similar manner but differently from the second flash positions close to the third flash (104%, 108%, 116%) and vice versa. However, the difference is not large as indicated by the regression coefficient of 0.08.

Individual results between participants varied but, on average, saltation was achieved when the second flash was presented in a reverse condition. In control conditions, when stimuli were presented at a slower speed, participants were able to report the positions of the second flash almost accurately, indicating the importance of stimulus duration and timing. One would expect these reverse conditions to cause a ‘break’ in the flow of the stimuli since such positions go against previous priors which dictate a moving object should occur in sequence. Yet, for illusion conditions, participants perceived the second flash to occur somewhere between the first and the last flash position, even when the second flash was presented near the third flash, akin to responses in Experiment 1.

One possible explanation for saltation in the reverse condition could be the brain adapting to the visual crowding of stimuli; perceiving the second flash closer to the midpoint is the average position of where the brain believes it should be (Whitney & Levi, 2011). Crowding combined with higher speed presentation of the second flash are not typical stimuli our eyes receive on a day-to-day basis. As there is a lack of “prior” knowledge of such stimuli, the flashes are reconstructed into a pattern that makes sense: the flash is reported at the midpoint because it is consistent with real-world stimuli where fast-moving objects tend to travel in a linear sequence (Seriès & Seitz, 2013). This can be combined with both post-and predictive effects to determine where the brain believes the second flash position should occur. For example, if the second flash occurred to the right of the first flash, and the third flash appeared to the left of the first flash, the brain would process the second flash to occur as some point left of the first flash, as this is the most logical order that a moving object would follow.

Another possible explanation for the perception of saltation under illusion conditions is that either the first or third flash—depending on the second flash position—was misperceived as the second flash. This misperception of flash order led to a rearrangement of the perceived sequential order of the flashes. If the second flash was presented in reverse (e.g., −8%) to the first flash, then the actual second flash will be perceived to be the first flash, and the actual first flash (which is closer to the midpoint) will be shifted perceptually near the midpoint. This hypothesis still plays into the idea of a postdictive effect under illusion conditions since a perceived positional shift still occurred through retrospective interpretation of the three-flash event.

It is also interesting to note in preliminary tests, that some individuals verbally reported that they saw only two flashes (only responses of participants who perceived all three flashes were used for data analysis) while undertaking illusion conditions where the second flash was presented out of bounds. These individuals usually reported only perceiving one flash approximately at the same position as the first flash, and a second (or third) flash approximately at the same position as the third flash. However, when the second flash was presented at the midpoint in illusion conditions, they reported seeing all three flashes. It is possible in out of bounds conditions, the brain disregards the second flash because it occurred out of sequence at such a high presentation speed, that there was not enough time to process it. It is also possible that the first or third flash was disregarded, and these individuals perceived the first two or the latter two flashes due to high presentation speeds.

## Experiment 3: Second Flash out of Linear Alignment

Experiment 2 revealed that saltation can occur even if flashes are not presented in a spatially sequential order. However, for both Experiments 1 and 2, flashes occurred in linear alignment, a parameter that is constant in saltation illusions. Would saltation be possible if the position of the second flash was presented outside of alignment? To test this, in Experiment 3 the second flash was presented at the midpoint between the first and the third flash—which is in alignment with the fixation point—but at different vertical locations. We hypothesized that the second flash would be perceived at a position in horizontal alignment with the first and the third flash positions because simple linear movement would be favored as a postdictively reconstructed path for high-speed object motion.

### Methods

#### Participants

Preliminary tests were conducted to ensure participants can perceive three flashes throughout the conditions. Potential participants were exposed to some conditions where the second flash was completely out of alignment (such as in the upper corner of the screen, or close to the fixation point) to observe if their attention was focused on the task and not automatically clicking a certain point. Only individuals who could perceive three flashes were allowed to take part. A total of 17 participants took part in the experiment (2 male, 14 female, and 1 who did not disclose their gender, ages ranging from 23–40). Four individuals partook in both Experiments 1 and 2, while six had participated in Experiment 2 only. Participants gave written consent and were compensated for their time, except for a co-author who also took part in the experiment.

#### Procedure

**Figure 5.**
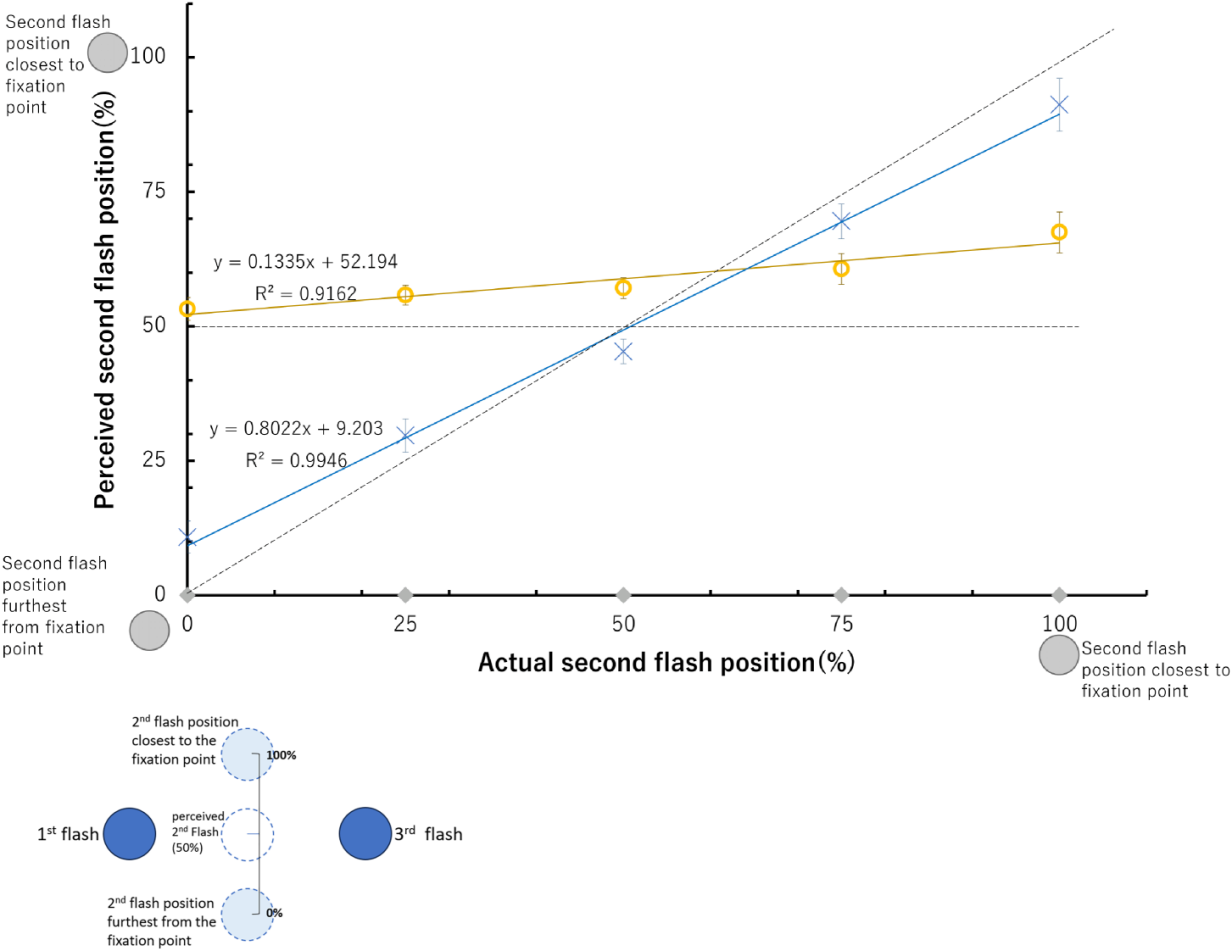
Perceived proportion of second flash positions relative to the first and third flash. Grey diamonds indicate actual flash positions, yellow circles (participant responses in illusion condition) and blue x's (participant responses in the control condition) correspond to the same positions. The value of 0 represents the second flash at the farthest position from the fixation point and 100 represents the second flash position closest to the fixation point. The value of 50 represents the horizontally aligned position with the first and third flashes. The dashed diagonal line indicates values where an illusion is not perceived, while the horizontal dashed line indicates saltation perceived at the midpoint. Inset shows how participant perception is interpreted into a percentage value.

**Figure 6.**
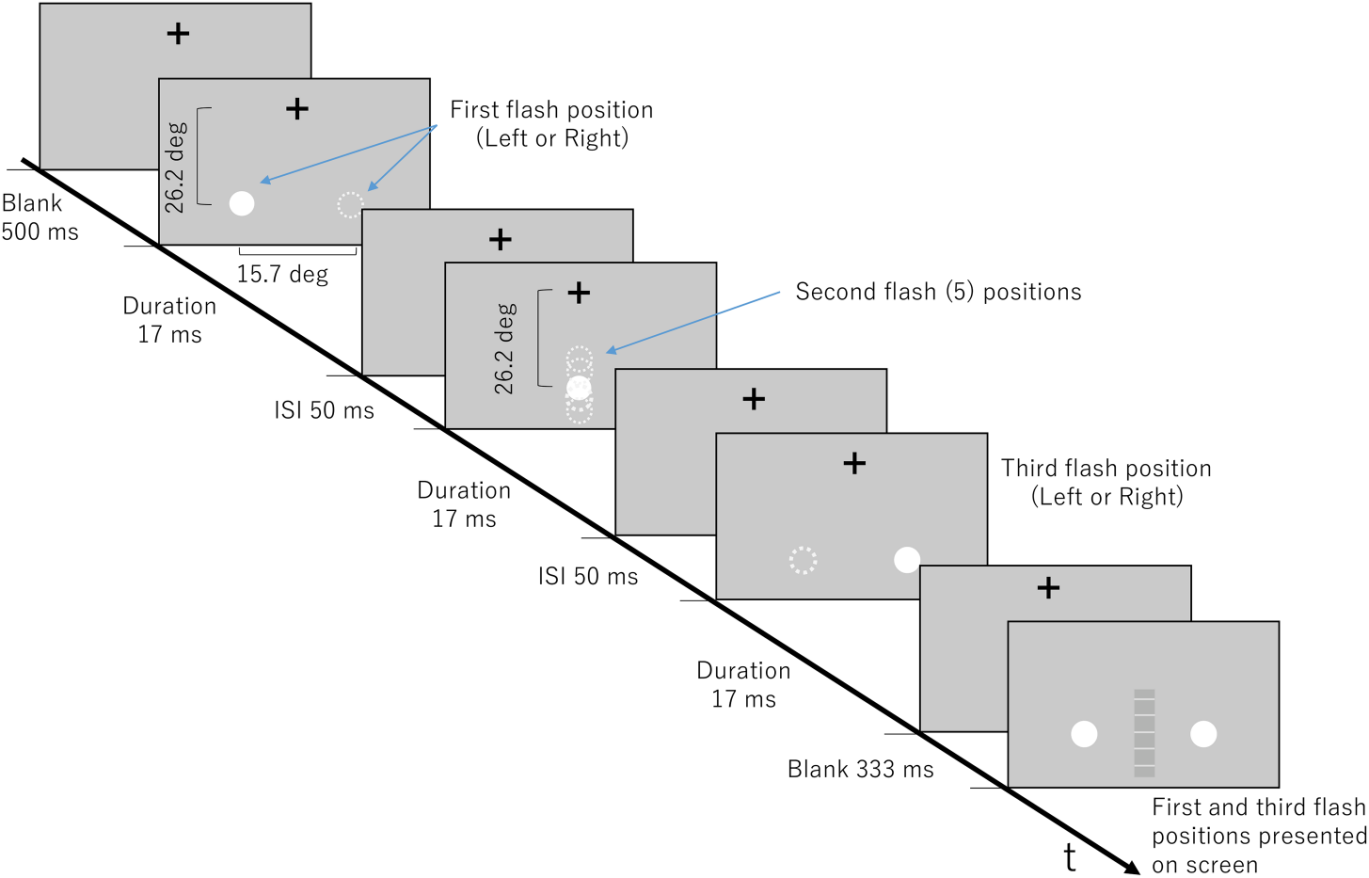
Experiment 3 stimulus parameters for the illusion condition. Experimental display that gives an example of stimuli moving in the left to right direction, indicated by solid white circles. Dashed white circles indicate other possible positions the flash could occur during the indicated time. The last screen displays the scale-response option; a free-response option looks the same but without the scale.

The same experimental set up as Experiments 1 and 2 was utilized. Position of stimuli shifted 2.6 deg vertically closer to the fixation point than previous experiments. The position of the second flash occurred at five possible locations vertically along the midpoint of the first and the last flash. These second flash positions were located 30.15 deg (0% in Figure 5,), 28.18 deg (25%), 26.2 deg (50%); 24.22 deg (75%), and 22.22 deg (100%) below the fixation point. Pilot tests revealed that some individuals did not see the second flash at the vertical midline between first and third flash, but still perceived the second flash to occur in linear (horizonal) alignment with the first and last flash. Others consistently saw the second flash to occur along the vertical midline. Thus, based on practice trials, participants were assigned a vertical scale response screen or, a free-response screen where they were able to click at any location on the screen (Figure 6).

The scale's physical appearance was the same as Experiments 1 and 2 but was rotated 90 degrees so that it appeared at the midpoint of the first and third flash positions, on the same vertical line as the fixation point (Figure 6). The scale had the same light gray markings that did not correspond to any of the second flash positions, except for a mark at the center that represented the midpoint of the first and third flash positions. Selecting this mark would indicate the participant perceived the second flash to occur in horizontal alignment with the first and third flash. For scale-responders, the scale would appear simultaneously with two white circles that represent the first and third flash positions after three flashes occurred (Figure 6); they were free to click anywhere on the scale. For free-responses, after a trial occurred, only the two white circles would appear on the screen and participants were free to click at any point on the screen. Participants were instructed to use the white circles to help indicate where they perceived the second flash. Only vertical values of the free-responses were used for data analysis.

There were five second flash positions, presented in the left or right direction, creating a total of 10 conditions. Participants underwent the 10 conditions six times, under a control and an illusion setting, creating a total of 120 trials per participant. The illusion condition presented flashes with an ISI of 50 ms and a SOA of 67 ms, while the control condition had an ISI of 1000 ms and SOA of 1033 ms.

### Results and Discussion

One participant's (P17) results were on average the same in control conditions (perceiving the second flash at the vertical midpoint) as well as in illusion conditions (perceiving the second flash around 30.15 deg below the fixation point). Grubb's test for outliers was employed. It was found that P17's responses on 3 out of the 10 conditions under the illusion parameters were outliers. P17 verbally reported seeing the three flashes form an arch in illusion conditions and perceiving the flashes mostly in a straight line in control conditions. Based on the outlier test and the inability to perceive the flashes in their approximate positions in control conditions, P17's responses were not included in the final data report. The following results are those of 16 participants only.

Out of the 16 participants, only three perceived the second flash to occur along the vertical midline between the first and their flash positions and were given the scale response. The rest used the free-response option. For easier data analysis, the y-components of free-responses were converted to scale values. Due to the low number of scale responders, their responses were combined the free-responses and analyzed together.

Figure 5 displays the results of the participant responses of how they perceived the second flash in the illusionary and control conditions both in the right and the left direction. Throughout all illusion conditions, the 16 participants misperceived the second flash to occur somewhat in alignment with the first and last flash. Second flash positions farther from the fixation point (i.e., below 50% position) were more likely to be reported to occur exactly at the midpoint of the second flash (Figure 5).

The main effects of direction, the position of the second flash, and the timing (control and illusion) as well as their interaction effects on perception of the second flash were analyzed using a three-way repeated measures ANOVA. As the violation of sphericity was indicated by the Mauchly's test, Greenhouse-Geisser's epsilon was used to adjust the degree of freedom. Just as in Experiments 1 and 2, the direction the flashes were presented was not significant (*F*(1,15) = 0.3233, *p *= .5780, *ηp^2 ^*= 0.0211). The effect of presentation timing (*F*(1, 15) = 15.055, *p *= .0015, *ηp^2^*= 0.5009) was significant. The main effect of the vertical positions of the second flash (*F*(1.48, 22.15) = 110.8029, *p *< .0001, *ηp^2^*= 0.8808) was highly significant. The interaction between the vertical flash position and the control and illusion condition (*F*(2.38, 35.68) = 96.0262, *p *< .0001, *ηp^2^*= 0.8649) was also highly significant. The difference in regression coefficients (0.1335 for the illusion condition vs. 0.8022 for the control condition) indicates that the perceived vertical position of the second flash did not vary much when the flashes are presented at high speeds, versus slower speeds, depending on the changes in the actual vertical position of the second flash.

Shaffer's MSRBP found significant differences for the second flash perceptions at all positions in control conditions. Under control conditions, participants were able to locate the approximate position of the second flash. Under illusion conditions, MSRBP showed significant differences between the second flash position closest to the fixation point (100%) and each flash position; at 75% (*p* = .0013, adjusted *p* = .0122), 50% (*p* = .0012, adjusted *p* = .0122), 25% and 0% (*p* = .0017, adjusted *p* = .0122). Flash positions closer to the fovea would be less likely to be perceived in linear alignment and therefore more likely to be perceived accurately even when presented at high speeds. A significant difference was found between the flash position at 75% and at 0% (*p* = .0066, adjusted *p* = .0396) as well. Flash positions at and below the horizontal midpoint were perceived in a similar manner.

Under the illusion condition, the farther the second flash was from the fixation point, the more likely the participant would select the second flash to occur close to the vertical midpoint of the first and third flash. This may be attributed to the fact that objects presented farther from the fovea are harder to detect spatially when the presentation is brief. Because there is limited information or spatial ambiguity, the brain processes the stimuli with the least information to occur in a pattern that makes the most sense. While flashes closer to the fixation point are closer to the fovea and therefore should be more likely to be perceived in the correct position, these flash positions were still misperceived to occur farther from their physical position. The speed of stimulus presentation possibly allowed second flash positions to be misjudged to occur close to the midpoint in the illusion conditions, although not as close to the midpoint as second flash positions further from the fixation point. Thus, reflecting top-down processing is more dominant in experimental setups such as the VSI, consistent with previous perceptual studies (Dijkstra et al., 2017).

This experiment is limited as it only presents the second flash along the vertical midpoint of the first and the third flash. Future experiments can approach the vertical shift in combination with the parameters of Experiments 1 and 2. However, the results of Experiment 3 are very promising for future saltation illusions, indicating that linear presentations of stimuli are not necessary to achieve saltation.

## General Discussion

This study explored a classic illusion by varying the position of the second flash in relation to the first and third, introducing it in the same position as the third flash, a reverse position, and outside of linear alignment, demonstrating the brain's remarkable ability to construct meaningful interpretations from visual stimuli. These modifications to the traditional saltation experiment not only challenge previous constraints but also highlight the flexibility of human perceptual systems. These findings align with the CRE conditions outlined by Geldard and Sherrick (1972), providing evidence that novel positions of the second stimulus can still elicit perceptual hopping effects if the duration and ISI are optimally set. Our methodology, following Geldard's (1976) work, involved using a minimal stimulus configuration of three flashes to dissect the complexities of low-level and high-level processing effects, a significant leap from prior studies where the first and second flashes were collocated and where flash stimuli are presented in alignment.

The observed effects from these experiments cannot be solely attributed to low-level motion signals, particularly when considering the altered positions of the second flash that do not align with expected motion directions. The perception of the second flash at a midpoint opposite the motion direction in Experiments 1 and 2, or beyond the third and/or first flash position in Experiment 2, suggests a reversal in motion perception that challenges the motion-signal hypothesis. These phenomena, akin to the flash-grab effect (Cavanagh & Anstis, 2013), point towards a postdictive mechanism where the brain reconstructs the event's sequence after receiving signals, indicating a level of perceptual processing that goes beyond simple motion tracking. In Experiment 3, flashes are still presented in the left or right direction, with only the vertical position of the second flash changing between trials. Here, motion signals can explain the second flash shifting away from the horizontal midpoint, but not shifting down or up towards the vertical midpoint. A postdictive hypothesis may be plausible that the three positions of the flashes are assigned as a perceptually reconstructed event after three flash signals are received at the brain.

The interplay between attention and VSI is touched on in Experiment 3, where the illusion's intensity diminished for stimuli closer to the fixation point. Experiment 3 shows the possibility of an attentional affect, while also aligning with Geldard's (1976) observations that the VSI predominantly occurs in peripheral vision. This pattern is consistent with reports indicating that focused attention weakens similar illusions, such as the flash lag effect (Baldo et al., 2002; Sarich et al., 2007; Shioiri et al., 2010), where the unpredictable positioning of stimuli amplifies the illusion, suggesting a contrast to VSI's behavior where predictability of flash position change might intensify the illusion. Adamian and Cavanagh (2017) further elucidated that directed attention, especially when participants concentrate on specific aspects like a flash position, significantly alters the perception of illusions such as the Fröhlich effect. This insight, coupled with evidence that subsequent motion signals can correct misperceptions by revealing an object's true starting position (Eagleman & Sejnowski, 2007), points towards how attention and prediction of motion paths influence VSI. These findings pave the way for future inquiries into the role of attention in VSI, especially how the perception of flash sequences and the consequent illusions are affected by the focal point of attention and the predictability of motion.

Cognitive biases may also explain saltation between the three experiments. Past research on slow priors theorizes that the typical exposure to slow-moving objects does not perceptually prepare our brain to process fast-moving stimuli such as those in visual saltation experiments (Seriès & Seitz, 2013), which leads to the misperception of stimuli. The results of this study show that although there is a similar perception of such fast-moving stimuli peripherally, on average there are some individuals who process such stimuli differently, such as participants who were not able to process all three flashes. By applying the idea of how past experiences shape perception, it may be possible to train the senses to perceive fast-moving stimuli. Normal people who look down from a skyscraper report seeing people walking on the street as ants, while window cleaners who have been exposed to vision from extreme heights, do not report the same description (Gregory, 2015). This can be a clue on how saltation illusions can be “broken” or even strengthened. It would be interesting to test the illusion for a baseball player who is accustomed to viewing a ball in rapid motion.

Optimizing flash duration and ISI is essential for inducing the saltation effect in VSI, highlighting the effectiveness of these parameters and their role as a limitation in our study. The need to adjust these parameters became apparent when the initial settings from Experiment 1 were insufficient to elicit the desired saltation effect in later experiments, necessitating varied timing adjustments to accommodate different second flash position conditions. This underscores the relationship between stimulus positioning and timing requirements to achieve visual saltation, indicating the importance of tailoring these variables specifically for each experimental setup, especially when diverging from traditional stimulus presentations. Our findings further suggest that regardless of the second flash's spatial position, when three flashes are presented in quick succession, the perceived location of the second flash tends to be near the center of the first and third flashes. This indicates that stimuli presented within a short temporal frame are processed collectively as a single event, emphasizing the dual influence of timing on both motion detection and postdictive processing across our experiments. Such integrated perception implies a uniform expectation for the second flash's appearance between the first and third flashes, irrespective of its actual placement. This insight into how temporal adjustments can influence perceived spatial relationships within groups, without altering between-group perceptions, sets groundwork for future research to explore universal temporal parameters that might govern the perception of saltation and other spatiotemporal illusions.

Investigating novel second flash positions shows even more possibilities for the VSI. Successful saltation can be carried on in different presentation modes of the illusion, such as 2D or 3D rotation. However, the results of this study suggest that for these novel versions, customized parameters would be necessary for successful saltation. Future experiments can also utilize the novel second stimulus positions presented in this study using different sensory modalities; outcomes of such experiments can reveal nuances in the somatosensory cortex.

## References

[bibr1-20416695241254016] Adamian N. Cavanagh P. (2017). Fröhlich effect and delays of visual attention. Journal of Vision, 17, 3–3. 10.1167/17.1.3 28114485

[bibr2-20416695241254016] Asai T. Kanayama N. (2012). "Cutaneous rabbit" hops toward a light: Unimodal and cross-modal causality on the skin. Frontiers in Psychology, 3, 427. 10.3389/fpsyg.2012.00427 23133432 PMC3490328

[bibr3-20416695241254016] Baldo M. V. Kihara A. H. Namba J. Klein S. A. (2002). Evidence for an attentional component of the perceptual misalignment between moving and flashing stimuli. Perception, 31, 17–30. 10.1068/p3302 11971260

[bibr4-20416695241254016] Blankenburg F. Ruff C. C. Deichmann R. Rees G. Driver J. (2006). The cutaneous rabbit illusion affects human primary sensory cortex somatotopically. PLoS Biology, 4, e69. 10.1371/journal.pbio.0040069 PMC1382015 16494530

[bibr5-20416695241254016] Bremer C. D. Pittenger J. B. Warren R. Jenkins J. J. (1977). An illusion of auditory saltation similar to the cutaneous” rabbit”. The American Journal of Psychology, 90(4), 645–654. 10.2307/1421738 610449

[bibr6-20416695241254016] Cavanagh P. Anstis S. (2013). The flash grab effect. Vision Research, 91, 8–20. 10.1016/j.visres.2013.07.007 23872166 PMC5047291

[bibr7-20416695241254016] Dijkstra N. Zeidman P. Ondobaka S. van Gerven M. A. Friston K. (2017). Distinct top-down and bottom-up brain connectivity during visual perception and imagery. Scientific Reports, 7, 1–9. 10.1038/s41598-017-05888-8 28720781 PMC5516016

[bibr8-20416695241254016] Eagleman D. M. Sejnowski T. J. (2000). Motion integration and postdiction in visual awareness. Science, 287, 2036–2038. 10.1126/science.287.5460.2036 10720334

[bibr9-20416695241254016] Eagleman D. M. Sejnowski T. J. (2007). Motion signals bias localization judgments: A unified explanation for the flash-lag, flash-drag, flash-jump, and Frohlich illusions. Journal of Vision, 7, 3–3. 10.1167/7.4.3 PMC2276694 17461687

[bibr10-20416695241254016] Flach R. Haggard P. (2006). The cutaneous rabbit revisited. Journal of Experimental Psychology: Human Perception and Performance, 32, 717. 10.1037/0096-1523.32.3.717 16822134

[bibr11-20416695241254016] Fröhlich F. W. (December 1924). Über die Messung der Empfindungszeit. Pflügers Archiv für die Gesamte Physiologie des Menschen und der Tiere, 202, 566–572. 10.1007/BF01723521

[bibr12-20416695241254016] Geldard F. A. (1976). The saltatory effect in vision. Sensory Processes, 1, 77–86. 1029079

[bibr13-20416695241254016] Geldard F. A. Sherrick C. E. (1972). The cutaneous” rabbit": A perceptual illusion. Science, 178, 178–179. 10.1126/science.178.4057.178 5076909

[bibr14-20416695241254016] Goldreich D. Tong J. (2013). Prediction, postdiction, and perceptual length contraction: A Bayesian low-speed prior captures the cutaneous rabbit and related illusions. Frontiers in Psychology, 4, 221. 10.3389/fpsyg.2013.00221 23675360 PMC3650428

[bibr15-20416695241254016] Gregory R. L. (2015). Eye and brain: The psychology of seeing, Vol. 38. Princeton university press.

[bibr16-20416695241254016] Ito H. Kubo K. de Jesus S. A. M. (2023). Visual saltation illusion induced by flashes of subjective contours. i-Perception, 14, 1–13. https://doi.org10.1177/20416695231191241 10.1177/20416695231191241 PMC10422910 37575682

[bibr17-20416695241254016] Ito H. Ogawa M. Sunaga S. (2013). Evaluation of an organic light-emitting diode display for precise visual stimulation. Journal of Vision, 13, 6. 10.1167/13.7.6 23757510

[bibr18-20416695241254016] Khuu S. K. Kidd J. C. Phu J. Khambiye S. (2010). A cyclopean visual saltation illusion reveals perceptual grouping in three-dimensional space. Journal of Vision, 10, 26–26. 10.1167/10.14.26 21191136

[bibr19-20416695241254016] Lee J. Kim Y. Kim G. (2012). Funneling and saltation effects for tactile interaction with virtual objects. Proceedings of the SIGCHI Conference on Human Factors in Computing Systems, 3141–3148. 10.1145/2207676.2208729

[bibr20-20416695241254016] Lockhead G. R. Johnson R. C. Gold F. M. (1980). Saltation through the blind spot. Perception & Psychophysics, 27, 545–549. 10.3758/BF03198683 7393702

[bibr21-20416695241254016] Mackay D. M. (1958). Perceptual stability of a stroboscopically lit visual field containing self-luminous objects. Nature, 181, 507–508. 10.1038/181507a0 13517199

[bibr22-20416695241254016] Miyazaki M. Hirashima M. Nozaki D. (2010). The “cutaneous rabbit” hopping out of the body. Journal of Neuroscience, 30, 1856–1860. 10.1523/JNEUROSCI.3887-09.2010 20130194 PMC6633980

[bibr23-20416695241254016] Nijhawan R. (1994). Motion extrapolation in catching. Nature, 370, 256–257. 10.1038/370256b0 8035873

[bibr24-20416695241254016] Peirce J. W. Gray J. R. Simpson S. MacAskill M. R. Höchenberger R. Sogo H. Kastman E. Lindeløv J. (2019). PsychoPy2: experiments in behavior made easy. Behavior Research Methods, 51, 195–203. 10.3758/s13428-018-01193-y 30734206 PMC6420413

[bibr25-20416695241254016] R Core Team (2021). R: A language and environment for statistical computing. R Foundation for Statistical Computing, Vienna, Austria. https://www.R-project.org/.

[bibr26-20416695241254016] Sarich D. Chappell M. Burgess C. (2007). Dividing attention in the flash-lag illusion. Vision Research, 47, 544–547. 10.1016/j.visres.2006.09.029 17173948

[bibr27-20416695241254016] Seriès P. Seitz A. R. (2013). Learning what to expect (in visual perception). Frontiers in Human Neuroscience, 7, 668. 10.3389/fnhum.2013.00668 24187536 PMC3807544

[bibr28-20416695241254016] Shim W. M. Cavanagh P. (2006). Bi-directional illusory position shifts toward the end point of apparent motion. Vision Research, 46, 3214–3222. 10.1016/j.visres.2006.04.001 16774774

[bibr29-20416695241254016] Shimojo S. (2014). Postdiction: Its implications on visual awareness, hindsight, and sense of agency. Frontiers in Psychology, 5, 196. 10.3389/fpsyg.2014.00196 24744739 PMC3978293

[bibr30-20416695241254016] Shioiri S. Yamamoto K. Oshida H. Matsubara K. Yaguchi H. (2010). Measuring attention using flash-lag effect. Journal of Vision, 10, 10–10. 10.1167/10.10.10 20884475

[bibr31-20416695241254016] Shore D. I. Hall S. E. Klein R. M. (1998). Auditory saltation: A new measure for an old illusion. The Journal of the Acoustical Society of America, 103, 3730–3733. 10.1121/1.423093 9637053

[bibr32-20416695241254016] Trojan J. Stolle A. M. Kleinböhl D. Mørch C. D. Arendt-Nielsen L. Hölzl R. (2006). The saltation illusion demonstrates integrative processing of spatiotemporal information in thermoceptive and nociceptive networks. Experimental Brain Research, 170, 88–96. 10.1007/s00221-005-0190-z 16328290

[bibr33-20416695241254016] Whitney D. Cavanagh P. (2000). Motion distorts visual space: Shifting the perceived position of remote stationary objects. Nature Neuroscience, 3, 954–959. 10.1038/78878 10966628

[bibr34-20416695241254016] Whitney D. Levi D. M. (2011). Visual crowding: A fundamental limit on conscious perception and object recognition. Trends in Cognitive Sciences, 15, 160–168. 10.1016/j.tics.2011.02.005 21420894 PMC3070834

